# Bacterial extracellular vesicles as players in biofilm dynamics and community interactions

**DOI:** 10.1093/femsre/fuag047

**Published:** 2026-09-16

**Authors:** Victoria Namiganda, Jeffrey W Schertzer

**Affiliations:** Department of Biological Sciences, Binghamton University, Binghamton, NY 13902, United States; Binghamton Biofilm Research Center, Binghamton University, Binghamton, NY 13902, United States; Department of Biological Sciences, Binghamton University, Binghamton, NY 13902, United States; Binghamton Biofilm Research Center, Binghamton University, Binghamton, NY 13902, United States

**Keywords:** extracellular vesicles, outer membrane vesicles, biofilms, biofilm development, biofilm matrix, bacterial communities

## Abstract

The ability to use vesicles to deliver cargo safely across distance is a valuable strategy that appears to be universal across all life. The ability of prokaryotes to accomplish this is a relatively new idea, but one that is expanding rapidly. In this review, we will describe what bacterial extracellular vesicles (bEVs) are and where they come from. We will also summarize what has been learned about their biogenesis and function through foundational planktonic studies. Using this as a backdrop, we will then contextualize new and exciting discoveries made as the focus of the field has shifted to studying bEVs in more complex multi-species and biofilm communities. We will explore how, when and where bEVs are produced in biofilms and how the circumstances of their formation can affect communication, matrix remodeling, community metabolism, organization, and defense. The boundless versatility of bEVs makes them essential contributors to bacterial community life and also offers great potential for exploitation as applied biologics.

## Teaser

This review will describe the current understanding of bacterial extracellular vesicle (bEV) formation and function, mostly gleaned from planktonic studies, before introducing new advancements at the frontier of bEV contributions to the development and maintenance of biofilms.

## Introduction

Vesicular transport was once believed to be exclusive to eukaryotes. Textbooks described transport between organelles and highlighted this as a distinguishing feature absent from prokaryotes. With the growth of the exosome field in the early 2000s, interest exploded in the field of *extracellular* vesicular trafficking, generating a great deal of enthusiasm for the study of vesicle-based secretion. This interest spilled over into the prokaryotic literature and elevated a small field that had documented the budding of vesicles from the surface of Gram-negative bacteria to produce what became known as outer membrane vesicles (OMVs). Since that time, we have come to appreciate that cells from all domains of life produce extracellular vesicles and that these vesicles perform myriad functions of great importance.

Perhaps it should not have come as a surprise that vesicular transport is a universal feature of life. The advantages of such systems are great. Bacteria possess many systems to secrete effector molecules into the environment (Rapisarda and Fronzes 2018), but traditional contact-dependent and contact-independent systems have specific drawbacks. Contact-dependent systems provide the opportunity for excellent target selection and direct delivery of effectors but necessarily have strong distance limits. Traditional contact-independent systems can extend reach to more distant targets but suffer from the challenges of dilution and interference/destruction by the environment. Transport using bacterial extracellular vesicles (bEVs), recently dubbed secretion system type zero (Guerrero-Mandujano et al. 2017), provides a paradigm that solves each of these weaknesses quite elegantly. By packaging cargo into vesicular delivery vehicles, bacteria create a system where effectors can be delivered over great distances while still maintaining effective concentrations at the point of contact. Further, the cargo within the vesicle will be protected throughout the journey, whether the target is near or far. The advantages of precise target selection can also be maintained because the surface of the vesicle is organized at the time of release and can contain any receptor or ligand present on the surface of the producing cell. In short, cargo trafficking using vesicles provides an extremely effective and versatile platform for bacteria to interact with other cells and their environment.

**Figure 1:**
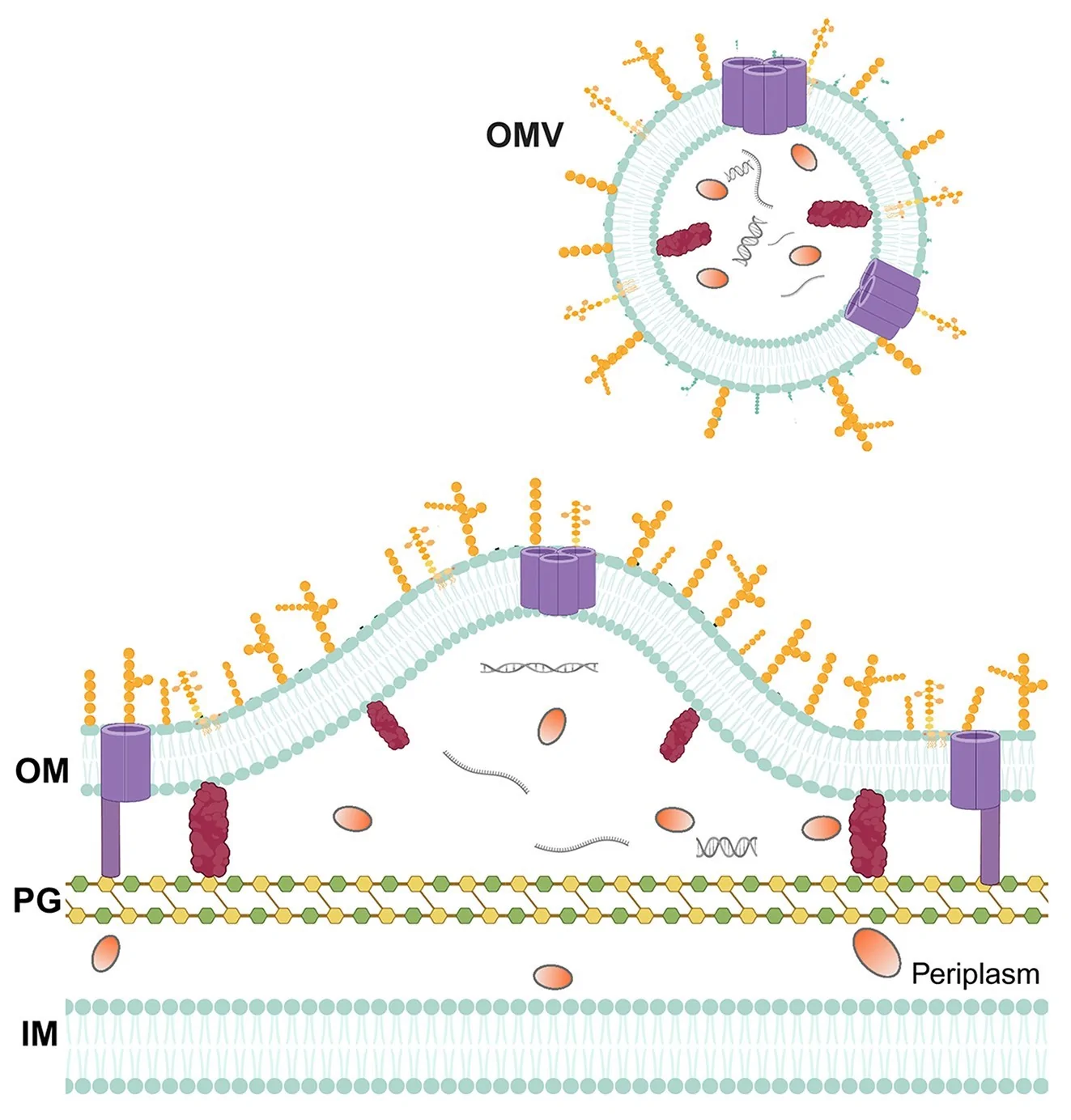
Anatomy of an OMV. The first type of bEVs to be rigorously characterized were the OMVs produced by Gram-negative bacteria. The vesicles bud from the OM of cells, so typically contain OM-embedded proteins and material that is captured from the underlying periplasm. OMVs typically resemble the OM in lipid identity, lipopolysaccharide content, and physical properties such as buoyant density. Created using biorender.com.

What is a bEV? That question has become increasingly complex over time. The first discovered and best characterized bEVs are derived from the outer membrane of Gram-negative bacteria and largely contain OM and periplasmic proteins (OMVs—Fig. 1) (Vella and Schertzer 2015, Avila-calderón et al. 2021). However, studies on Gram-positive bacteria and investigations into alternate mechanisms of biogenesis have uncovered different types of bEVs that are composed of different types of membrane and may contain cargo from different cellular compartments (Toyofuku et al. 2023).

Despite the relatively recent rise in interest around bEVs, their existence has been documented for at least 50 years. In the 1960s, Work and colleagues characterized what they termed “extracellular lipoglycopeptide” that was excreted into the supernatant by *Escherichia coli* (Bishop and Work 1965). Chemical characterization of this material confirmed it was composed of lipopolysaccharide from the outer membrane (Taylor et al. 1966), and electron microscopic analysis showed that the material consisted of “blebs” that had been extruded from the cells (Knox et al. 1966). Since that time, extracellular vesicles have been observed in cultures of nearly every organism tested. Perhaps the first documentation of bEVs in biofilms came in 1975 when Halhoul and Colvin made specific note of “vesicle-like” structures in electron micrographs of gingival plaque (Halhoul and Colvin 1975) and suggested they might play a role in bacterial adhesion.

Work on Gram-negative OMVs has dominated the field until recently. Foundational studies rigorously characterized the constituents of the vesicles in order to confirm their provenance. OMVs were demonstrated to resemble the OM much more than the IM in terms of lipid composition (Hoekstra et al. 1976, Horstman and Kuehn 2000, Kato et al. 2002), fatty acid composition (Hoekstra et al. 1976), buoyant density (Hoekstra et al. 1976, Renelli et al. 2004), and the ratios of phospholipid (PL):protein, 3-deoxy-d-manno-oct-2-ulosonic acid (KDO):protein, and lipopolysaccharide (LPS):PL (Hoekstra et al. 1976, Katsui et al. 1982). Examination of marker enzymes including glucose-6-dehydrogenase (Gankema et al. 1980), NADH oxidase (Hoekstra et al. 1976, Horstman and Kuehn 2000, Renelli et al. 2004), lactate dehydrogenase (Hoekstra et al. 1976), and succinate dehydrogenase (Hoekstra et al. 1976, Florez et al. 2017, Horspool and Schertzer 2018) confirmed that OMVs were composed almost exclusively of OM and did not arise simply through cellular disintegration. Recent work in Gram-positives (Toyofuku, Nomura et al. 2019, Toyofuku, Schild et al. 2023) and some Gram-negatives (Turnbull et al. 2016) have shown that, although bEVs may largely arise from dedicated and regulated mechanistic pathways, cell lysis can contribute to their formation, especially under certain circumstances. By comparing the amount of OM protein released into the supernatant vs. retained in cells, it has been estimated that *E. coli* releases up to 5% (Gankema et al. 1980) and *Pseudomonas aeruginosa* between 0.75% and 2.5% of their total outer membrane as OMVs (Bauman and Kuehn 2006).

It is clear that vesicular transport contributes greatly to the biology of all lifeforms. While eukaryotic cells possess a host of dedicated protein systems for membrane bending, scission, and fusion, homologous systems are conspicuously absent from bacteria. As such, increasing our understanding of the formation and function of bEVs could shed light on exciting evolutionary questions about how sophisticated transport systems developed from more primitive modes of packaging and delivery. With this review, we aim to familiarize the reader with the general contours of what is known about bEV formation and function (mostly gleaned from studies of planktonic bacteria). We will then describe recent advancements that are beginning to orient the field toward community interactions and the role of vesicle transport in bacterial biofilms.

## Insights from planktonic studies

In order to contextualize recent discoveries about bEVs in bacterial communities and biofilms, it is necessary to provide a description of what is generally known about how they are formed and what they do. Until very recently, this knowledge has come almost exclusively from studies of planktonic bacteria. Here, we will organize the existing understanding into a few broad categories to describe bEV formation and function.

### Membrane vesicle formation

#### Integrity of the cellular envelope

**Figure 2:**
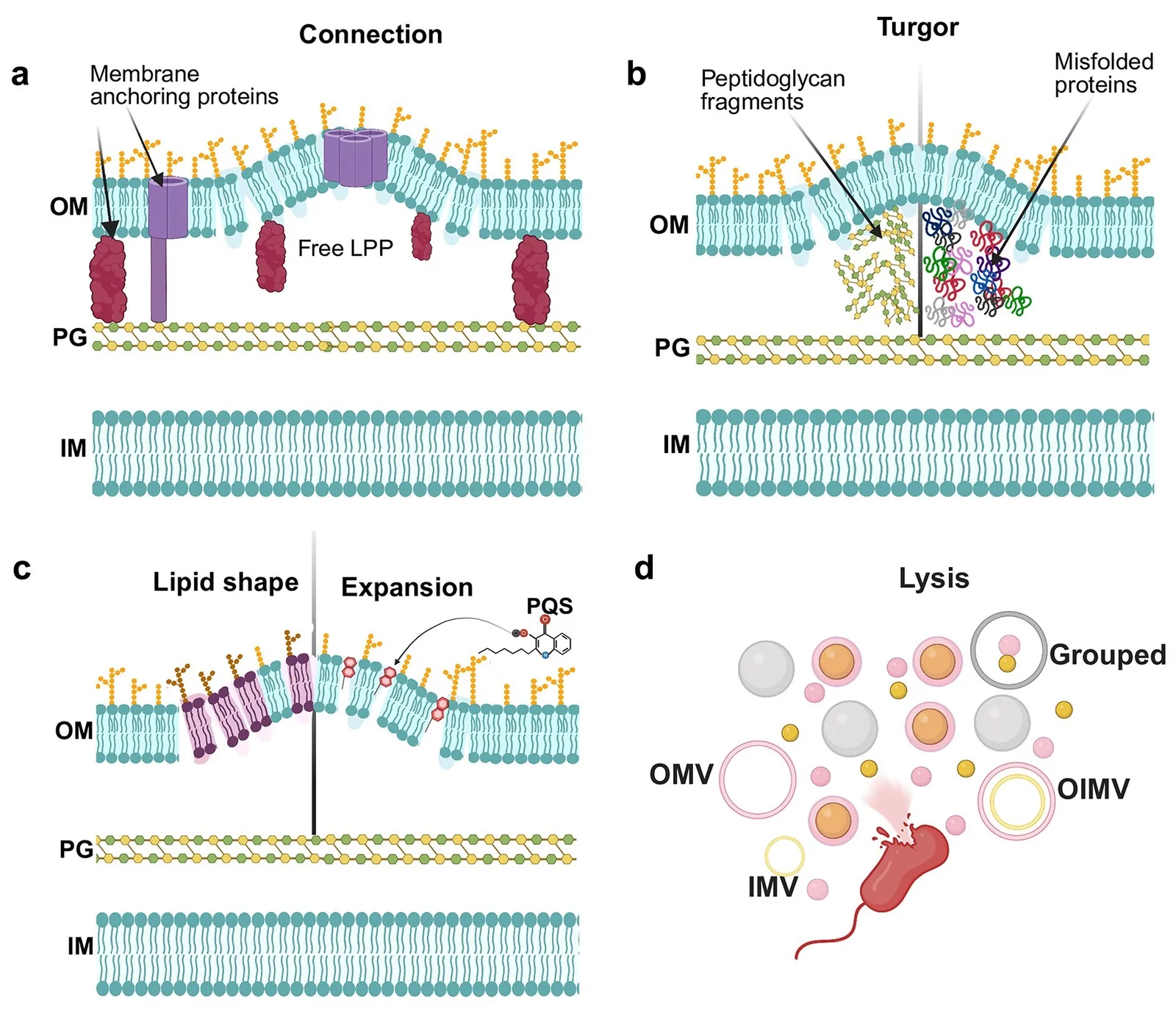
Mechanisms of bEV biogenesis. Studies of bEV formation in planktonic cultures have yielded considerable information about the ways in which vesicles can be formed. (a) Differences in the density of OM-PG connections, mediated by specific proteins, can dictate how many bEVs are formed and from which regions of the cell. (b) Cell wall maintenance and turnover can produce PG fragments that accumulate to increase periplasmic turgor that contributes to OM bulging (left). Cell envelope stress caused by multiple sources, including the accumulation of misfolded proteins, can induce a similar effect (right). (c) Direct induction of OM curvature can be achieved by alteration of lipid shape or identity in the OM (left), or by intercalation of curvature-inducing molecules like the Pseudomonas quinolone signal (PQS) (the Bilayer Couple Model—right). (d) A variety of vesicle types can be created when a cell lyses. Cellular contents are then trapped within vesicles by membrane fragment recircularization. Created using biorender.com.

One of the earliest explanations for the release of membrane material by bacterial cells into the environment centered around transient disconnection of the membrane from the cell (Fig. 2a). In fact, the idea that bEVs exist at all was first dismissed as an artifact of cell lysis (more on that below) and still comes under some criticism today (Coelho and Casadevall 2019). As with most of the bEV field, the majority of biogenesis models focus on the Gram-negative cellular envelope, and for this section we will focus our discussion on that context as well. One straightforward model for OMV biogenesis is that fragments of the OM are sometimes lost during routine remodeling of the cell envelope during the normal life cycle of a cell. The most clear-cut example of this “passive” type of vesicle release comes from electron microscopic analysis of cells undergoing division (Burdett and Murray 1974, Weigand et al. 1976, Deatherage et al. 2009). It was reported that loose portions of the OM bulge out during septum formation and are often shed as vesicles. However, the fact that the authors also saw blebs released from the cell body meant that a septum-only mechanism could not fully explain OMV formation. A more generalized idea began to emerge that transient release of OM anchoring proteins might explain OMV production at any location along the cell. This was supported by very early protein profiling of purified OMVs. When the protein content of OMVs was compared to that of the OM, it was evident that OM-PG anchoring proteins such as Braun’s lipoprotein were selected against as vesicle cargo (Hoekstra et al. 1976, Wensink and Witholt 1981). This stands to reason because a region of the OM can’t be released as an OMV if it remains connected to PG. Consistent with this, Braun’s lipoprotein that did make its way into OMVs was discovered to be predominantly in the PG-unlinked form (Wensink and Witholt 1981). Intentional knockout of anchor proteins such as Braun’s lipoprotein or the Tol–Pal complex have been confirmed to result in hypervesiculation (Wessel et al. 2013, Takaki et al. 2020), as have other mutations that result in reduced lipoprotein binding to PG (Schwechheimer et al. 2015).

An interesting deviation from the pattern of OM-PG anchor protein exclusion from OMVs involves the widely conserved OmpA family of proteins. Members of this family have well-documented roles anchoring the OM to PG (Rawling et al. 1998, Park et al. 2012), and their deletion results in hypervesiculation (Song et al. 2008, Moon et al. 2012, Wessel et al. 2013). However, members of this family are known to be abundant in OMVs across species (Lee et al. 2008, Wurpel et al. 2014, Alves et al. 2015). This suggests that OmpA-family proteins are present at the site of OMV formation and are packaged into vesicles despite their ability to bind PG. This paradox can likely be explained by a unique physical feature of these proteins. They are capable of adopting two different conformations in the OM: a two-domain conformation where the C-terminus extends into the periplasm to interact with PG, and a one-domain conformation where the entire protein inserts into the membrane to form a porin (Nikaido 2003). Recent work from our laboratory demonstrated that PG association of OprF (OmpA homolog) in *P. aeruginosa* was critical to prevent hypervesiculation in this species, and that the conformational distribution swings from majority two-domain (anchored) in the OM to majority one-domain (released) in OMVs. Thus we proposed the “Latch Model” where conformational dynamics in OmpA-family proteins govern local OM-PG connectedness and therefore help define which regions of the OM will bleb into OMVs (Mathur et al. 2026).

#### Periplasmic/envelope homeostasis

Another area that has been explored as a mechanism of OMV formation is that of periplasmic and OM maintenance (Fig. 2b). The PG cell wall is constantly being turned over during growth and division of the cell. The presence of cell wall components in OMVs has suggested that PG remodeling may be particularly active near the sites of OMV biogenesis (Knox et al. 1966, Work et al. 1966). Not only could this potentially break OM-PG linkages, but the accumulation of PG degradation products was proposed to create excess turgor in the periplasm, leading to bulging of the OM and the initiation of OMV formation (Zhou et al. 1998) (Fig. 2b—left side). Subsequent studies investigating mutants affected in PG turnover (Hayashi et al. 2002), structure (Schwechheimer et al. 2014), and dynamics (Schwechheimer et al. 2015) have strengthened support for the role of PG remodeling in OMV biogenesis.

Other types of envelope stress have also been tied to OMV biogenesis. Environmental conditions such as pH and temperature were shown to trigger OMV production. As were assaults on the envelope by antibiotics (Walker and Beveridge 1988, Kadurugamuwa and Beveridge 1995), cationic peptides (Macdonald and Kuehn 2013), chelators (Marvin et al. 1989), oxidizers (Sabra et al. 2003), and hydrophobic compounds (Baumgarten, Sperling et al. 2012, Baumgarten, Vazquez et al. 2012). These may all involve some aspects of chemical weakening of the envelope, but Kuehn and colleagues set out to determine if dedicated cellular pathways might connect the disparate inputs to a response of increased OMV release. *Escherichia coli* transposon screens aimed at identifying genes involved in OMV biogenesis highlighted hits in the σ^E^ envelope stress pathway (McBroom et al. 2006, Kulp et al. 2015). The authors speculated that impairment of the σ^E^ pathway would lead to the accumulation of misfolded or mistargeted proteins in the periplasm and that this could stimulate OMV formation to remove the unwanted material (Fig. 2b—right side). By engineering a peptide to mimic a misfolded protein and targeting it to the periplasm, they went on to show that this mechanism was plausible (McBroom and Kuehn 2007). Recent discoveries have also highlighted the contributions to OMV biogenesis of an important anti-sigma factor in the Bacteroidota (Pardue et al. 2024). Organisms in this phylum express a unique dual-membrane spanning anti-sigma factor that is degraded upon activation by signals in the environment or membrane perturbation. This degradation then causes the release of the sigma factor into the cytoplasm to stimulate OMV biogenesis, further strengthening a connection between envelope sensing and the production of bEVs.

The ability to eject patches of the OM as OMVs provides a useful mechanism to accomplish membrane remodeling as well as cell wall turnover. When a cell requires a rapid change in Lipid A composition, perhaps due to environmental change, it has been proposed that OMVs package and release the undesirable lipids in order to make room for newly synthesized Lipid A. Bonnington and Kuehn measured the distribution of Lipid A in cellular OM vs. OMVs after rapid environmental shift and discovered that specific lipid chemotypes were retained with the cell while others were jettisoned in the OMVs (Bonnington and Kuehn 2016). Thus, OMV production appears to be connected to Lipid A/LPS turnover in bacteria. Once discarded lipids leave one cell, they may be taken up by other cells, leading to advantages for the community (Zingl et al. 2020). This is an example of a case where the drivers of bEV production may contribute directly to community function (more on this below).

#### Curvature manipulation via lipids or small molecules

The process of OMV biogenesis must necessarily begin with the induction of curvature in the OM (Fig. 2C). It is well understood that lipids, proteins, and other molecules of particular shapes have a preference for more or less curved membranes. This is also true of the LPS that comprises the outer leaflet of the bacterial OM. Although it has been shown that the O-antigen portion of LPS is not required for OMV production in multiple species (Nguyen et al. 2003, Haurat et al. 2011, Murphy et al. 2014), Haurat *et. al*. investigated whether different Lipid A chemotypes were preferentially packaged into *Porphyromonas gingivalis* OMVs (Haurat et al. 2011). They discovered that de-acylated Lipid A, which has a shape that prefers positively curved membranes, was preferentially sorted into OMVs. Similar sorting has been observed in *Salmonella* (Bonnington and Kuehn 2016). Also working in *Salmonella*, it was shown that overexpression of the Lipid A deacylase PagL increased both OMV production and packaging of the deacylated Lipid A into vesicles (Elhenawy et al. 2016). These results strongly suggest that building regions of the OM that prefer positive curvature will encourage OMVs to form (Fig. 2c—left side).

Membrane curvature can also be manipulated by the insertion of membrane active small molecules. Perhaps the most deeply tested biochemical mechanism of OMV biogenesis centers around the ability of the Pseudomonas Quinolone Signal (PQS) to induce membrane curvature in *P. aeruginosa*. PQS is a secreted quorum sensing signal (Pesci et al. 1999) that was discovered to be trafficked in the membranes of OMVs when it was asked how such a hydrophobic molecule could be transferred between cells (Mashburn and Whiteley 2005). What was unexpected about this discovery was that PQS also stimulated the formation of the vesicles into which it was packaged, and did so through a biochemical mechanism that was separate from its quorum sensing function (Mashburn and Whiteley 2005). Subsequent biophysical and structure-activity studies suggested that a strong interaction between PQS and Lipid A was the driving force behind the mechanism (Mashburn-Warren et al. 2008, 2009). To explain this phenomenon, we developed the Bilayer-Couple Model of OMV biogenesis, which posits that hydrophobic PQS inserts into the outer leaflet of the OM where it resists flip–flop due to its strong affinity for Lipid A (Schertzer and Whiteley 2012). Accumulation of PQS in the outer leaflet then leads to expansion of the outer leaflet relative to the inner leaflet, which causes a mismatch in leaflet tension that is alleviated through the development of membrane curvature (Fig. 2c—right side). We and others have since supported the model by showing that PQS must be exported to be effective (Florez et al. 2017) and that PQS stimulates OMV formation in non-Pseudomonas species as well (Tashiro, Ichikawa, Nakajima-Kambe et al. 2010, Horspool and Schertzer 2018). Most recently, we extended structure-activity analysis using experiments and molecular dynamics simulation to show that the PQS ring nitrogen is critical for PQS-Lipid A interaction, as well as the previously characterized hydroxyl group and alkyl chain, and that PQS energetically prefers the OM outer leaflet owing to increased hydrogen bonding to Lipid A (Gopal et al. 2025). Interestingly, Sinha et al. (2019) showed in *Citrobacter rodentium* that covalent modification of Lipid A phosphates by ethanolamine reduces OMV production. If this organism operates a similar mechanism to *P. aeruginosa*, then it is likely that phosphate conjugation blocks critical interactions with an as-yet-undiscovered PQS-like molecule produced by *C. rodentium*.

The Bilayer-Couple model has also been invoked to explain the initiation of vesicle budding due to phospholipid imbalance in the OM. Roier et al. (2016) discovered that if proper phospholipid recycling is disrupted in Gram-negative bacteria, this leads to hypervesiculation. The VacJ/Yrb and homologous Mla-type systems (Malinverni and Silhavy 2009, Baarda et al. 2019, Davies et al. 2019) are responsible for retrograde PL transport from the OM. The authors suggest that degraded ability to remove PL from the OM results in leakage of PL into the outer leaflet, which then induces membrane curvature in a manner similar to PQS. Consistent with this, OMV preparations recovered from VacJ/YrbE mutant strains were measured to contain increased PL (Roier et al. 2016). It is noteworthy that outer leaflet localization of the increased PL was not demonstrated, and that contamination of OMV preps by inner membrane vesicles released by potentially increased cell lysis would give the same results. Nevertheless, the model is consistent with results from the PQS system and provides the potential to describe a general mechanism for bEV production owing to the wide conservation of Mla systems across bacteria.

#### Cell lysis

A major concern in the bEV field is whether observations are confounded by cellular debris originating from cell lysis. When cells rupture, membrane sheets spontaneously re-circularize to form vesicles (Fig. 2d) that might be difficult to distinguish from bEVs that arise through dedicated mechanistic pathways. In 2016, Turnbull et al. (2016) released a study proposing explosive cell lysis as a program for the production of bEVs that at least was subject to some genetic regulation. They showed that certain cells in a *P. aeruginosa* population would spontaneously round up and then lyse, releasing bEVs as a product. The autolytic program was connected to an endogenous prophage endolysin, and therefore set the stage for discussion of programed cell death as a contributor to bEV formation. We will discuss this topic again later in the context of bEV formation in biofilms.

With the lysis door opened, and once extracellular vesicles began to be consistently observed in Gram-positive cultures, the bEV biogenesis paradigm had to be completely re-examined. Gram-positive organisms lack an OM and possess thick PG cell walls between the plasma membrane and extracellular environments. Both of these facts presented hurdles to establishing the legitimacy of bEVs produced by Gram-positives (Coelho and Casadevall 2019). While strong criticisms remain that Gram-positive bEVs are nothing more than cellular debris, or at least must have significantly different importance/functions than Gram-negative bEVs, great strides have been made to understand how they are produced (Briaud and Carroll 2020). Several triggers have been associated with Gram-positive bEV production, including two-component regulatory systems (Resch et al. 2016), phenol soluble modulins (Wang X et al. 2018), endogenous polyketides (Hoefler et al. 2017), and prophage endolysins (Toyofuku, Cárcamo-Oyarce et al. 2017, Andreoni et al. 2019). Regardless of how bEV production is triggered in Gram-positive organisms, the mechanism normally leads to cell death, either by explosive cell lysis or “bubbling cell death” (Toyofuku, Cárcamo-Oyarce et al. 2017, Toyofuku, Nomura et al. 2019) where the cytoplasmic membrane protrudes through large ruptures in the cell wall to produce bEVs before the cell dies, leaving behind a ghost cell. However, Jeong et al. (2022) recently observed bEV formation in *Staphylococcus* spp. using super–resolution stochastic optical reconstruction microscopy and claim to see evidence of a blebbing mechanism that gives rise to bEV “precursors” between the cell membrane and PG. These bEV precursors would still have to find their way out of the cell through PG degradation, which would likely also lead to cell death.

It is important to note that the vesicles produced by lysis mechanisms are distinct in composition and cargo from those produced by non-lytic mechanisms, especially for Gram-negative bEVs. Explosive lysis vesicles can be composed of OM only (OMVs), OM and IM (OIMVs), or only cytoplasmic membrane (CMVs or IMVs) (Toyofuku, Nomura et al. 2019, Toyofuku, Schild et al. 2023). The vesicles can also be unilamellar or multilamellar, or they can consist of multiple smaller vesicles within a larger vesicle (“grouped”) (Fig. 2d). Because these vesicles are produced by cell lysis and then reformed, they can also contain cargo from any cellular compartment.

### Membrane vesicle function

Because their cargo is so diverse, and can vary with time and environment across species, bEVs have been associated with a huge number of important bacterial processes. Here, we will survey illustrative examples in four general categories to give a basis for what types of functions may be relevant to community or biofilm interactions that will be discussed further below. As with bEV biogenesis, the vast majority of functional studies have been carried out using Gram-negative OMVs, so our discussion will focus there with Gram-positive examples included where appropriate.

#### Competition

Some of the first clues to the function of bacterial OMVs came from analysis of truly remarkable electron micrographs captured by Beveridge and coworkers as the bEV field was picking up in the 1990s. These images captured OMVs released from *P. aeruginosa* associating with the cell envelopes of *E. coli* and *S. aureus*. The vesicles then fused or ruptured, respectively, to release their contents and cause degradation of the cell wall at the point of contact (Kadurugamuwa and Beveridge 1996, 1999). This led to the idea that OMVs served as offensive weapons in the eternal conflict between bacteria for space and resources, leading to the term “predatory vesicles” (Kadurugamuwa and Beveridge 1996, Li Z et al. 1996). The Beveridge group characterized the presence of phospholipase, protease, hemolysin, alkaline phosphatase, and peptidoglycan hydrolase activity present in OMVs purified from *P. aeruginosa* (Kadurugamuwa and Beveridge 1995). Interestingly, and owing to the connection of PG turnover to OMV biogenesis described above, it was noted that OMVs were most effective at degrading the cell wall of non-self target bacteria that contained the same chemical type of PG as the OMV donor species (Li Z et al. 1998). This makes perfect sense if autolysins involved in PG turnover are picked up and packed into OMVs and then delivered to neighboring cells. Recent work by Chen et al. (2022) identified specific PG hydrolases present in *P. aeruginosa* OMVs and discovered that individual hydrolases were often dispensable for lytic activity, concluding that the bactericidal effect of bEVs may depend on the synergistic action of several enzymes. Lytic effects of bEVs have also been reported by others studying *Myxococcus* and *Lysobacter* species (Vasilyeva et al. 2008, Evans et al. 2012). It is now common to test OMV potency against competing bacteria using disk diffusion assays as one might for antibiotic susceptibility (Mashburn and Whiteley 2005, Cooke et al. 2019).

The use of OMVs as agents of competition extends beyond bacterial targets and crosses kingdom barriers. Afoshin et al. (2020) studied OMVs released by *Lysobacter capsica* and confirmed that the vesicles delivered a suite of proteases that “digested Staphylococcal cells with high efficiency”. Purified vesicles also lysed several other species of Gram-positive bacteria, fungus and yeast. Interestingly, the antifungal activity was found exclusively within the OMV fraction, while some antibacterial activity remained in the supernatant after the vesicles were removed. Similarly, Wang et al. (2025) described the antifungal activity of Myxococcal OMVs against the fungus *Verticillium dahlia*. bEVs are also effective killers and modulators of higher eukaryotes, but that will be further discussed under “Virulence”, below.

It is important to note that it is not only the enzymes packaged within OMVs that can kill or inhibit competing organisms. In addition to the lytic enzymes identified in *P. aeruginosa* OMVs, Mashburn and Whiteley discovered that solvent-extractable quinolones trafficked in vesicles could also kill *Staphylococcus epidermidis* on their own (Mashburn and Whiteley 2005). Here, it was demonstrated that an ethyl acetate extract of *P. aeruginosa* OMVs was nearly as effective at clearing *S. epidermidis* lawns as the purified whole vesicles. Violacein is another example of a small molecule poison trafficked in OMVs, this time by *Chromobacterium violaceum*. Several groups have demonstrated that this hydrophobic antibiotic is trafficked in OMVs and that purified *C. violaceum* vesicles are bactericidal against several Gram-positive target species (Batista et al. 2020, Choi et al. 2020). OMVs harvested from mutants in the violacein biosynthetic pathway lost the ability to kill, solidifying violacein as the active agent.

#### Virulence

bEVs are now well understood to traffic toxins and modulatory factors to higher eukaryotes. This makes them potent effectors of host-microbe interactions in a large number of systems. The existence of such transfer, and the mechanisms for how it occurs, took a large step forward when Bomberger et al. (2009) demonstrated that OMVs from *P. aeruginosa* could fuse with the plasma membrane of human cells to deliver multiple virulence factors directly into the cytoplasm. The investigators demonstrated OMV fusion to airway epithelial cells by labeling OMVs with a fluorescent rhodamine dye that self-quenches at high concentration. Vesicle fusion with the target cell allowed the lipophilic dye to dilute into the bulk plasma membrane and become fluorescent. OMV fusion was found to be dependent upon lipid rafts and N-WASP-initiated actin assembly. Most interestingly, the CFTR inhibitory factor (cif) toxin was found to be 17 000-fold more potent when delivered by OMVs vs. as purified protein, strongly highlighting the effectiveness and physiological relevance of vesicle-based toxin delivery. To further strengthen this point, Zingl *et. al*. showed that cholera toxin delivered in OMVs was equally effective at increasing c-AMP levels in target intestinal epithelial cells after caveolin-mediated endocytosis. Being delivered in a vesicle, however, greatly increased the resistance of the toxin to external protease and consequently allowed for its toxic potency to persist longer in the intestinal milieu (Zingl et al. 2021).

In addition to Bomberger *et. al*., several other groups have documented OMV fusion and direct delivery of cargo to mammalian cells (Demuth et al. 2003, Rompikuntal et al. 2012). In contrast, others have observed that OMVs are internalized into host cells by various endocytic routes (Kesty et al. 2004, Furuta et al. 2009, Amano et al. 2010). Rather than modulating CFTR transport like cif or c-AMP levels like cholera toxin, several reports have documented direct killing of eukaryotic cells in a manner reminiscent of the interbacterial competition described above. Demuth et al. (2003) studied the dental pathogen *Aggregatibacter actinomycetemcomitans* and showed that OMVs from this organism delivered a lytic leukotoxin to HL60 cells by direct membrane fusion. Treatment of the host cells by purified OMVs resulted in incorporation of leukotoxin into the plasma membrane followed by lysis. The fact that the HL60 cells became anti-leukotoxin antibody reactive in the presence of lysis inhibitors and at sublethal leukotoxin concentrations supported the conclusion that delivery was through direct membrane fusion. Evidence that OMVs can also be toxic to insect cells was provided when McMahon et al. (2012) injected *Serratia marcescens* OMVs into the haemocoel of *Galleria mellonella* (greater wax moth) larvae. Injection of 10 ng KDO equivalents of OMVs resulted in near eradication of the larval population in 24 h. In contrast, the same amount of purified LPS had only a small effect. These studies demonstrate that, depending upon cargo and conditions, OMVs can cause disease in animal hosts ranging from dysregulation to death.

The involvement of bEVs in disease is not limited to animal hosts, however. A number of plant pathogens also exert their effects through the use of bEVs (reviewed in Rudnicka et al. 2022). *Xylella fastidiosa* provides an interesting example of a case where a pathogen may employ bEVs for a purpose other than direct delivery of virulence factors. During the infection cycle, *X. fastidiosa* must convert from a firmly attached biofilm form in the foregut of the insect vector to a non-adhesive and “exploratory” form to rapidly disseminate through the xylem of the plant. Ionescu *et. al*. discovered that the exploratory form of the pathogen overproduces OMVs (apparently under quorum sensing control). These OMVs then bind to and block potential attachment sites on the xylem vessel wall, thereby inhibiting cell attachment as the pathogen rapidly spreads throughout the plant (Ionescu et al. 2014). Feitosa-Junior et al. (2019) later characterized the proteome of *X. fastidiosa* OMVs and showed that they contain typical virulence factors such as lipases, adhesins, proteases, porins, and pectin lyases. So, *X. fastidiosa* OMVs also likely participate in virulence through more traditional means. Proteomic analyses have also implicated OMVs as virulence factor delivery systems in other plant pathogens such as *Xanthomonas campestris* (Sidhu et al. 2008, Solé et al. 2015) and *Pseudomonas syringae* (Chowdhury and Jagannadham 2013).

#### Protection

Owing to their similarity with the bacterial outer surface, bEVs can be used to protect the mother cell by acting as decoys or sacrificial targets to avoid threats. Elements of this idea have been seen since at least the early 1990’s when Pettit and Judd investigated serum susceptibility of *Neisseria gonorrhoeae* in the presence or absence of OMVs (Pettit and Judd 1992). They reported that exogenous addition of OMVs from both serum susceptible and serum resistant strains protected cells from serum killing. OMVs from serum resistant strains were significantly better at protection. The authors hypothesized that the OMVs were binding serum antibodies away from the bacterial cells, and that the OMVs from resistant strains contained more or better antigens for this purpose. Subsequent studies have narrowed this effect down to sequestration of complement proteins. Tan et al. (2007) observed that *Haemophilus influenzae* cells were protected from serum killing if supplemented with *Moraxella catarrhalis* OMVs. These authors found that the protective effect stemmed from the ability of *M. catarrhalis* OMVs to bind C3 complement protein and keep it away from the *H. influenzae* cells. Similarly, Lindholm et al. (2020) found that *A. actinomycetemcomitans* OMVs displaying proteins OmpA1 and OmpA2 could protect *ompA1^−^ ompA2^−^* serum susceptible strains. Once again, this effect was due to sequestration of complement proteins by the OMVs. The multifunctional OprF protein of *P. aeruginosa* has also been shown to bind C3 complement protein (Mishra et al. 2015), giving evidence that the complement decoy strategy is widespread.

Complement proteins are not the only threats that can be neutralized by OMVs to protect the mother cell. When exploring obstacles to phage therapy against *Vibrio cholerae*, Reyes-Robles et al. (2018) discovered that OMVs produced by that bacterium had the capacity to protect against phage binding to cells. The authors presented remarkable cryo-electron tomograms showing binding of the tail fibers of multiple phage types to OMVs. Augustyniak *et. al*. presented similar results for the phage protective effects of *P. aeruginosa* OMVs, though they went further to ask whether OMVs could transfer phage receptors from one cell population to another (Augustyniak et al. 2022). Although OMVs from susceptible strains were seen to associate with the LPS-negative resistant strains by FACS, no transfer of receptors was measured to make the recipient cells sensitive. A more robust methodology to confirm integration of the OMVs into the cell outer membrane by fusion would help advance this interesting question.

OMVs have also been documented to protect cells against antimicrobial peptides and other antimicrobial compounds. Manning and Kuehn examined the protective role of OMVs against polymyxin B, colistin and phage using both a hypervesiculating *E. coli* mutant and OMV titrations (Manning and Kuehn 2011). OMVs provided immediate protection against each treatment but did not result in the acquisition of resistance, suggesting that the mechanism was once again threat adsorption as a decoy membrane. The generality of this mechanism was further demonstrated when OMVs were also shown to protect the yeast *Candida albicans* against polymyxin B (Roszkowiak et al. 2019). Traditional small molecule antibiotics do not escape inactivation by OMVs either, though the mechanism of inactivation is different in this case. Several studies have shown that degradative enzymes packaged within OMVs work to degrade antibiotics before they can reach the mother cell (Schaar et al. 2011, Kulkarni et al. 2015, González et al. 2016, Kim et al. 2018). The question of how enzymes packaged into the lumen of OMVs can affect antibiotic concentrations in the external environment was answered by our own work that showed the OprF porins of *P. aeruginosa*, while >90 closed in the OM of cells to act as an OM-PG anchor, are nearly exclusively open when exported in OMVs (Mathur et al. 2026). This means that OMVs would be highly permeable to small molecules (<3400 Da (Nestorovich et al. 2006)), allowing degradative enzymes to work efficiently to destroy antibiotics while remaining protected within the OMV.

Finally, bEVs can help protect the producing cell through modulation of the host immune response. Mechanisms to accomplish this are much more diverse than for threat avoidance as described above (Charpentier et al. 2023). bEVs can of course activate the immune response owing to LPS (Shah et al. 2012) and PG (Kaparakis et al. 2010) carried with them, but they have also been observed to manipulate the response in more subtle ways to benefit the producing cells. Gingipains carried in *Porphyromonas gingivalis* OMVs degrade LPS receptor CD14 from macrophages, resulting in a greatly reduced immune response (Duncan et al. 2004). *Legionella pneumophila* OMVs induce specific macrophage miRNA expression to make the host cells more permissive to bacterial replication (Jung et al. 2016). OMVs containing superantigen released by *M. catarrhalis* specifically activate B cells to direct the immune response away from the producing cells (Vidakovics et al. 2010). Thus, bEVs can be used in a number of clever ways to help protect against the host immune system.

#### Communication

Since bEVs are released from one cell and are known to interact/fuse with other cells in the population, it is natural that they would be used to facilitate communication. The classical example of this is the PQS quorum sensing system of *P. aeruginosa* (Pesci et al. 1999, Gallagher et al. 2002). This system involves a secreted quinolone signaling molecule that binds to a receptor (PqsR) in the recipient cell to direct gene expression and cell behavior. The PQS system integrates into an overlapping QS hierarchy in *P. aeruginosa* that controls several virulence and competitiveness phenotypes (Rampioni et al. 2016, Miranda et al. 2022). For example, co-culture of *P. aeruginosa* and *S. aureus* in a dialysis membrane chamber inserted into the peritoneal cavity of a rat resulted in PQS-dependent lysis of *S. aureus* by *P. aeruginosa* as a means to acquire nutrients (Mashburn et al. 2005). Uniquely for a QS signal at the time, PQS was discovered to be trafficked between cells in OMVs (Mashburn and Whiteley 2005). Because of its low solubility in water and strong interactions with Lipid A in the outer membrane (Mashburn-Warren et al. 2008, Gopal et al. 2025), PQS is believed to be inserted in the membrane of the OMVs that carry it. Mashburn and Whiteley (2005) also showed that exogenous addition of purified OMVs, as well as synthetic PQS, could restore PQS-dependent phenotypes in a PQS-null mutant. Hydrophobic Acyl-homoserine lactone signals have been reported to be trafficked in OMVs as well (Toyofuku, Morinaga et al. 2017). Long-chain cis-2 unsaturated fatty acid QS molecules of the diffusible signal factor (DSF) family have also been found to associate with OMVs (Ionescu et al. 2016, Feitosa-Junior et al. 2019) and, like PQS, have been associated with their biogenesis (Ionescu et al. 2014, Devos et al. 2015). It is unclear from these reports whether the effect of DSF is based on signaling or is biophysical in nature like PQS, but it is clear that bEVs provide an efficient transport vehicle for bacterial signaling molecules.

Another way bacteria can “communicate” with one another is through horizontal gene transfer. bEVs have long been associated with this process. Extracellular DNA has frequently been found to co-purify with bEVs of both Gram-negative and Gram-positive bacteria (Dorward and Garon 1990, Kadurugamuwa and Beveridge 1995, Kolling and Matthews 1999, Rodriguez and Kuehn 2020). In many species, these DNA-containing vesicles have been successfully demonstrated to transfer genes into self and non-self species (Yaron et al. 2000, Domingues and Nielsen 2017, Tran and Boedicker 2019, Li P et al. 2022, Johnston et al. 2023). *Pseudomonas aeruginosa* OMVs were even reported to transport DNA into eukaryotic cells (Bitto et al. 2017). Small RNAs are also frequently identified as bEV cargo and can carry out important functions (Tsatsaronis et al. 2018). For example, Moriano-Gutierrez et al. (2020) showed that the SsrA sRNA packaged into OMVs of *Vibrio fischeri* is successfully transported into cells of its squid host, where it helps facilitate proper colonization and luminescence in the light organ. *Pseudomonas aeruginosa, S. aureus* , and other species have also been found to use bEVs to deliver sRNAs into host cells, where they modulate cytokine production and the immune response (Koeppen et al. 2016, Ahmadi Badi et al. 2020, Rodriguez and Kuehn 2020).

## Extracellular vesicles in bacterial communites and biofilms

### bEVs in biofilms

It is now widely accepted that bacteria predominantly live a biofilm community lifestyle in nature and that biofilms contribute significantly to human disease (Costerton et al. 1995, Costerton et al. 1999). The ability of biofilm communities to tolerate very high concentrations of antibiotics is frequently highlighted, but there are also other advantages for bacteria to live in (often highly diverse) groups. Recognizing that vesicular transport presents what might be a critical mechanism for bacteria to manage large communities, the bEV field is beginning to catch up to the times. In principle, any of the bEV functions described in planktonic studies (above) could be important in the biofilm context as well. The aim of this section is to highlight recent advancements where the roles of bEVs in biofilms have actually been tested, and to gather what insight we can about their contributions to this important mode of growth.

The most basic question one could ask is whether bEVs are present in biofilms at all. This question comes with some irony since important early evidence for the existence of bEVs came from electron micrographs of dental plaque (Halhoul and Colvin 1975). Nevertheless, this area of investigation has lagged behind the study of bEVs from planktonic cultures. Confirmation of the widespread presence of bEVs in biofilms came about first through continued and expanded electron microscopy studies. Terry Beveridge’s laboratory was responsible for much of this foundational work during a time when the idea of bEVs was still very niche. They took advantage of freeze-substitution techniques to produce detailed micrographs that carefully preserved the structures of the biofilm matrix (Hunter and Beveridge 2005). Schooling and Beveridge conducted an important survey of biofilms sampled from laboratory bioreactors, water drains, fish aquariums, industrial plants, and more (Schooling and Beveridge 2006). The analysis showed that bEVs were abundant in the biofilm matrix of both single-species *P. aeruginosa* biofilms and diverse mixed-species environmental biofilms. More recent microscopic analyses have confirmed this is true in many species (Palsdottir et al. 2009, Yonezawa et al. 2009, Baeza and Mercade 2020). Interestingly, bEVs were found to be more abundant per cell in biofilms than in planktonic cultures (Schooling and Beveridge 2006), with Palsdottir et al. (2009) remarking that in the matrix of *Myxococcus xanthus* biofilms “almost every conceivable space is filled with vesicles”.

### bEVs in biofilm development

#### Do bEVs build or break biofilms?

**Figure 3:**
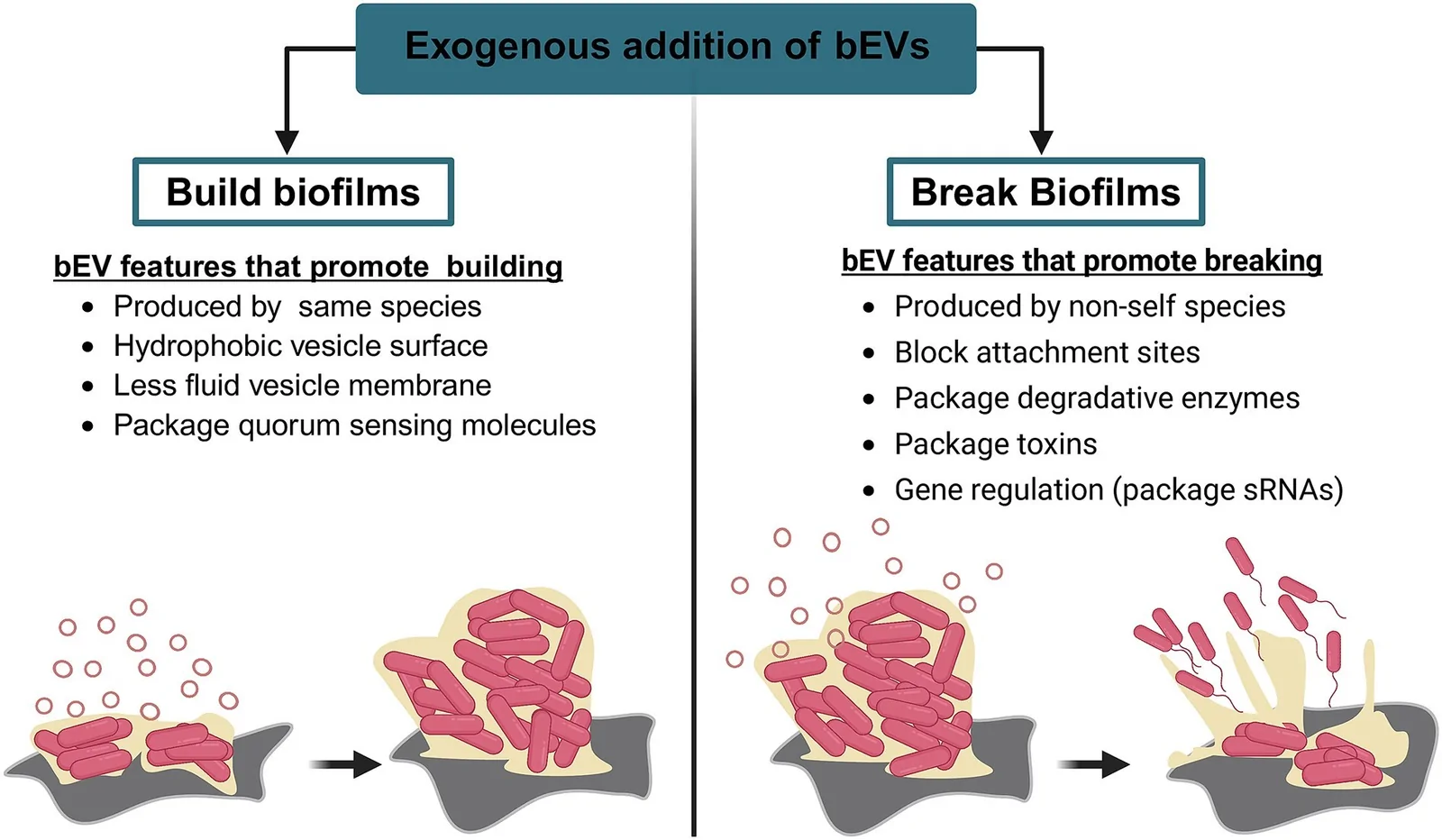
Do bEVs build or break biofilms? The effect that bEVs have on biofilm formation is nuanced and depends upon multiple factors. bEVs generally promote formation/stability of biofilms composed of the producing organism, though counterexamples do exist. When encountering biofilms composed of other species, bEVs may instead promote biofilm degradation. In each case, the function of bEVs has been associated with specific features or vesicle cargo. This topic represents an open question in the field and is an area ripe for further investigation. Created using biorender.com.

A natural place to begin the study of biofilm bEVs is to ask whether their production affects biofilm development. Likely due to nuances of experimental design, vesicle source, and species differences, the literature paints a surprisingly unclear picture on this topic (Fig. 3). In general, OMVs seem to promote biofilm formation for the species that produced them. Yonezawa et al. (2009) surveyed multiple strains of *Helicobacter pylori* for their ability to form biofilms. Most were poor biofilm formers, but one OMV-overproducing strain formed conspicuously robust biofilms. The authors followed up by showing that addition of excess OMVs from this strain further increased biofilm biomass. The same group later identified OMV cargo proteins that are associated with the increase in biofilm formation (Yonezawa et al. 2011, 2017). Baumgarten, Sperling et al. (2012) set out to investigate the role of *P. putida* OMVs in adaptive stress response. In response to several environmental shocks, *P. putida* responded by significantly increasing OMV production. Rapid vesicle release resulted in an increase in cell surface hydrophobicity, and enhanced the tendency to form biofilms. In a similar vein, Sabra et al. (2003) investigated the response of *P. aeruginosa* to variations in oxygen saturation. In concert with changes in A- and B-band LPS content in the OM, these authors found markedly increased OMV production as oxygen saturation increased, which in turn correlated with increased biofilm attachment to glass. It is interesting to note that the synthesis of PQS by *P. aeruginosa* requires molecular oxygen (Schertzer et al. 2010). Since PQS drives OMV formation in this organism (Mashburn and Whiteley 2005, Schertzer and Whiteley 2012), this may help explain the observed phenomenon. It is also consistent with the observations of Diggle et al. (2003) that exogenous addition of PQS to *P. aeruginosa* cultures promoted biofilm formation.

The previous examples detail how increased endogenous production of bEVs can increase biofilm formation, but experiments have also shown that exogenous addition of purified bEVs can do the same. Mozaheb et al. (2022) were interested in characterizing differences in membrane fluidity between planktonic vs. biofilm *P. aeruginosa* cells and their corresponding OMVs. They discovered that the membranes of biofilm cells were much less fluid than those of planktonic cells. Fascinatingly, exogenous addition of OMVs purified from biofilm cultures caused planktonic cell membranes to become rigid. Correspondingly, the authors found that incubation with OMVs increased biofilm formation—particularly if the OVMs were originally purified from biofilm cultures. Increased biofilm formation in response to bEVs may have potential impacts on disease transmission. Jiang et al. (2023) discovered that OMV production controlled by the SueI/SueR quorum sensing circuit in *Serratia ureilytica* had a strong effect on the ability of this organism to form into biofilm-like aggregates in the mosquito gut after a blood meal. This aggregate formation aids in gut colonization, which in turn reduces plasmodium transmission efficiency, potentially connecting bEV formation to anti-malarial strategies. Similar examples of bEV promoted biofilm formation have been documented in *Aeromonas* (Seike et al. 2021, 2025), *Acinetobacter* (Park J et al. 2021), *Vibrio* (Xiu et al. 2025), and *Shewanella* (Baeza and Mercade 2020) species.

Though bEV production has generally been seen to be positive for biofilm formation in the producing species, some outliers have been reported. As was described above, the case involving *Xylella fastidiosa* infection of plants is one where increased OMV production is used to block binding sites on the xylem wall and therefore prevent attachment and biofilm formation (Ionescu et al. 2014). In *Salmonella pullorum*, exogenous addition of purified OMVs was shown to reduce biofilm formation and decrease intestinal colonization (Lu et al. 2020). Mass spectrometry analysis hinted that enzymatic cargo within the OMVs may have contributed to biofilm degradation. In a similar fashion, Esoda and Kuehn (2019) found that OMVs from *P. aeruginosa* strains expressing the leucine aminopeptidase PaAP could disrupt established biofilms through their protease activity.

The phenomenon of bEVs promoting biofilm disruption is much more common when the vesicles encounter organisms different from the producer species. Wang Y et al. (2021) demonstrated that *Burkholderia thailandensis* OMVs could reduce *Streptococcus mutans* biofilm biomass, integrity and cell viability when administered exogenously. Much of this activity was ascribed to toxic quinolines and rhamnolipids delivered by the OMVs. In a similar analysis, Goes et al. (2021) showed that myxobacterial OMVs disrupt the biofilms of both Gram-negative and Gram-positive target species. The authors in this case hypothesized that the disruptive effect was the consequence of OMV packaged cystobactamid antibiotic synergizing with other OMV components to compromise the target biofilm matrix (bEV interactions with the biofilm matrix are described in more detail below). This idea is in line with the work of Cooke *et. al*. who showed that *P. aeruginosa* OMVs contain protease, lipase, and DNase activity (Cooke et al. 2020). A recent example of biofilm inhibition involving Gram-positive bEVs showed that *Staphylococcus epidermidis* bEVs have an inhibitory effect on *S. aureus* biofilms (Chunxing et al. 2025). These vesicles were thought to affect target biofilms by suppressing the cationic antimicrobial peptide resistance pathway. Used offensively against non-self bacteria, these strategies are reminiscent of examples described in the “competition” functional category above. Potential applications of these bEVs in treatment also strengthen the old Beveridge idea that bacterial vesicles may one day be used as “conceptually new antibiotics” (Kadurugamuwa and Beveridge 1996).

#### Cell lysis in biofilms

Many mechanisms have been proposed for the biogenesis of bEVs under different conditions and in different species (described above), but most studies have limited themselves to observing planktonic bEV formation. Questions remain, then, as to whether bEVs are produced by the same mechanisms in biofilms as in free swimming cells. The field has slowly begun to explore bEV production specifically in biofilms. A striking example of this is the mechanism of “explosive cell lysis” (Turnbull et al. 2016). This model for bEV production in *P. aeruginosa* was actually first described in interstitial biofilms, and it posits that activation of a prophage endolysin causes rounding and rupture of biofilm cells to release OMVs and eDNA. Squyres and Newman (2024) recently extended the use of real-time high-resolution microscopy to map cell lysis events throughout the *P. aeruginosa* biofilm. Interestingly, they showed that cell lysis is observable in biofilms but largely limited to a specific zone ~5 µm from the surface, whose position is dictated by oxygen and nutrient gradients.

#### PQS-induced OMVs in biofilms

Another major bEV biogenesis mechanism that has been specifically studied in the biofilm context is the Bilayer-Couple model involving PQS in *P. aeruginosa*. This model explains OMV formation as a result of membrane curvature induction triggered by PQS insertion into the OM (Schertzer and Whiteley 2012), but its operation in biofilms was questioned when the explosive cell lysis model was proposed (Turnbull et al. 2016). In that work, it was reported that a PQS-null mutant had no effect on OMV formation in the interstitial biofilm model that was used. It is important to note, however, that conditions within a hydrogel sandwiched between two closely opposed sheet of glass (the interstitial biofilm model) would likely be anoxic, and oxygen is required for PQS synthesis (Schertzer et al. 2010). This interpretation is supported by the fact that when *P. aeruginosa* was grown in the presence of oxygen, the endolysin-mediated effect disappeared, likely because it was swamped out by “regular” PQS-mediated OMV formation (Turnbull et al. 2016). We and others have also shown that *P. aeruginosa* makes very few OMVs under low oxygen conditions (Sabra et al. 2003, Schertzer et al. 2010).

Nevertheless, we set out to directly investigate PQS-induced OMV biogenesis in biofilms (Cooke et al. 2019). Using the colony biofilm model, we showed that loss of PQS production dramatically reduced OMV production, as it does for planktonic cultures. We did identify a small population of bEVs produced independently of PQS, and their size distribution was markedly larger than typical *P. aeruginosa* OMVs (Cooke et al. 2019). Comparison to cell debris generated through sonication suggested that this population likely did arise through cell lysis. We have since replicated these results using the 24-well plate biofilm model (unpublished). Overall, these analyses demonstrate that PQS is a major driver of OMV production in biofilms with access to at least mid-nanomolar concentrations of oxygen (the PqsH K_m_ for O_2_ is 520 nM (Schertzer et al. 2010)). However, there are likely multiple mechanisms at play simultaneously, and the PQS- and lysis-based mechanisms may even be intertwined. Contrary to our work (Cooke et al. 2019) and that of Turnbull et al. (2016), which both found that explosive cell lysis can occur independent of PQS production, others have reported a potential link. When the terminal steps of PQS biosynthesis were blocked, the accumulation of high concentrations of the pathway intermediate HHQ appeared to result in cell lysis in the centers of colony biofilms coincident with the activation of latent Pf4 prophage (D’Argenio et al. 2002, Giallonardi et al. 2023). Though no direct mechanism for HHQ induction of prophage was demonstrated, it is noteworthy that this phenomenon occurred only in biofilms.

#### bEV production is dynamic in biofilms

**Figure 4:**
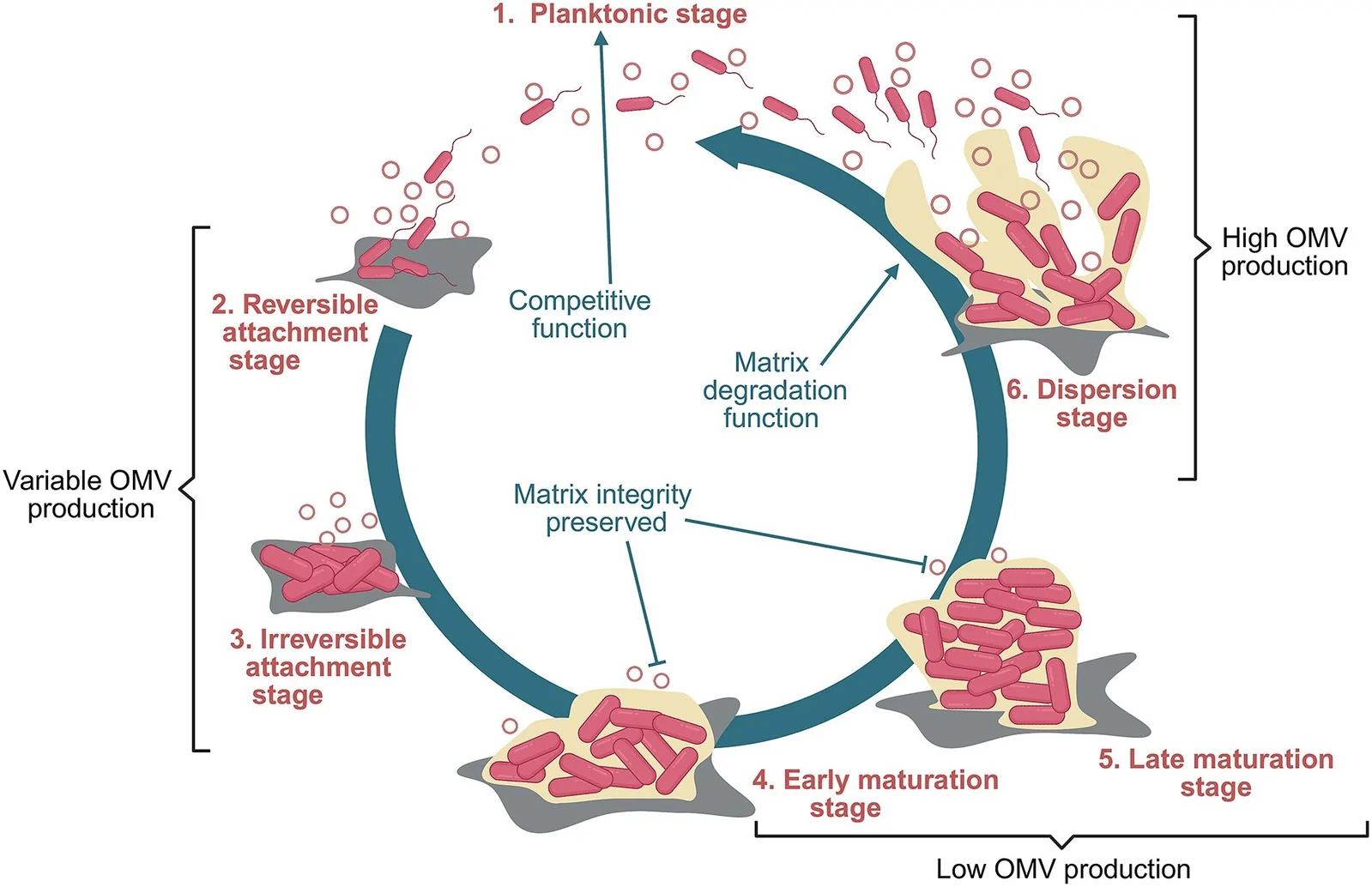
bEV production and function varies during the biofilm life cycle. The figure shows the classical progression through the six stages of biofilm development (numbered bold text, moving counter-clockwise from the top). Several reports describe how the number and nature of bEVs change as the biofilm transits through the developmental stages. As a consequence, we propose that bEVs may perform different functions at different stages of biofilm development. For example, *P. aeruginosa* OMVs are produced at a relatively high number during attachment, reduce to minimum production during maturation, and spike to maximum production during dispersion (text bracketing specific developmental stages). Enzymatic activity of OMVs tested from each stage suggest that the vesicles are best adapted for biofilm maintenance during maturation, matrix degradation during dispersion, and species-species competition during planktonic growth (central text pointing to specific stages). Created using biorender.com.

Biofilm formation is best understood as a developmental process, moving through defined stages and employing specific checkpoints (Sauer et al. 2002, 2022). Looking at planktonic growth through a similar lens, it is known that for some organisms bEV production changes throughout different growth stages. Tashiro, Ichikawa, Shimizu et al. (2010) investigated this for *P. aeruginosa* and found that OMVs varied in number, size, buoyant density, zeta potential, and protein content as cultures progressed through exponential to stationary phase. We were interested in discovering whether OMV production in *P. aeruginosa* biofilms followed a similar pattern. Using continuous flow biofilm tube reactors and marking the five stages of biofilm growth (Sauer et al. 2002), we observed that biofilm OMV production (normalized per cell) was highly dynamic across stages (Cooke et al. 2020). Production was variable at the earliest attachment stages, then diminished through the maturation stages, before accelerating markedly during the dispersion phase (Fig. 4). Notably, this pattern matched the dynamics of PQS production, which further supported PQS-induced biogenesis as an operative mechanism during biofilm growth. In fact, we noted that mutants lacking PQS-dependent OMV formation were deficient in biofilm dispersion, and that this phenotype could be complemented by both expression of *pqs* genes and exogenous addition of PQS itself (Cooke et al. 2020) (more on this below).

Physical characteristics of bEVs can also vary in biofilms. To date, it has been more common to compare biofilm bEVs to planktonic bEVs. Size differences were noted early, with Schooling and Beveridge (2006) observing bEVs recovered from lab grown and natural biofilms to be smaller on average than planktonic bEVs. This is interesting in light of our analysis of OMV size over time in *P. aeruginosa* biofilms. In a colony biofilm model, we showed that OMVs became larger as the community aged, and that vesicle size correlated with the ability to inhibit *S. epidermidis* growth (Cooke et al. 2019). Given that Tashiro also saw OMVs size increase over time in planktonic cultures (Tashiro, Ichikawa, Shimizu et al. 2010), this highlights the need for investigators to keep in mind *when* planktonic vs. biofilm bEVs are harvested if the goal is to compare them. This extends to other properties as well. Grande et al. (2015) examined bEVs from *H. pylori* and found a significant difference in surface charge between planktonic and biofilm vesicles. This could influence the binding of extracellular DNA and the ability of vesicles to associate with one another, two phenotypic changes that these authors also observed. Interactions of bEVs with the biofilm matrix are discussed in greater detail below.

#### Do bEV functions vary with developmental stage?

It is one thing to understand whether bEVs produced over time in a biofilm differ in number and physical properties, but a more central question is whether these differences might lead to alternate bEV functions depending upon when they were produced. An indirect way to test for this is to analyze the bEV proteome. Similar to the situation with physical measurements, this question has mostly been addressed by comparing planktonic vs. biofilm cultures, though some time-resolved studies have also been performed. At a global level, proteomic analysis in both Gram-negative (Toyofuku et al. 2012) and Gram-positive (Karched and Alkandari 2025) organisms have shown that bEV proteomes differ significantly between biofilm and planktonic growth modes. Zeroing in on *P. aeruginosa*, the Khursigara group performed an in-depth analysis of biofilm OMVs at different stages of development. First, they confirmed that biofilm OMVs have a distinct proteome compared to planktonic OMVs (Park AJ et al. 2014). Interestingly, biofilm OMVs differed more greatly from their planktonic counterparts than did biofilm cells. They followed this up with a comparative proteomic analysis of cells vs. OMVs in planktonic vs. biofilm cultures, each at three different timepoints (Park AJ et al. 2015). This analysis revealed that biofilm OMV content is distinct from the parent cell, varies over time, and shows evidence of selective cargo packaging. With varying numbers and different cargo profiles at different times, the rationale is established to propose that bEVs carry out different functions depending on the biofilm development stage.

When our group observed the connection between OMV number and dispersion in *P. aeruginosa* biofilms (Cooke et al. 2020), we asked whether the vesicles could be connected to this function. Using planktonic OMVs as proof of principle, we demonstrated that purified intact vesicles were capable of degrading proteins, lipids and DNA. Each of these macromolecules is a component of the biofilm matrix. We therefore proposed that a burst of OMV biogenesis (initiated by a preceding burst of PQS production) could serve to deliver degradative enzymes to the matrix to facilitate EPS breakdown and cell release (i.e. biofilm dispersion). After the development of protocols to purify OMVs from continuous flow biofilms at different stages of development, we set out to ask whether vesicles possessed different levels of matrix-degrading enzyme activity at different biofilm development stages. We found that dispersion phase OMVs had five-fold greater protease activity and 100-fold greater lipase activity than planktonic OMVs (unpublished observations). OMVs from the maturation phase had a different pattern. Protease activity was equal to planktonic OMVs, and lipase activity was intermediate between planktonic and dispersion OMVs (∼20-fold increased over planktonic). Interestingly, anti-Staphylococcal competitiveness was found to be greatest in the planktonic OMVs, consistent with the agar plate biofilm data (Cooke et al. 2019). This analysis evaluating actual enzymatic activity found in vesicles strongly supports the idea that OMVs carry out different functions depending upon developmental stage (Fig. 4).

### bEVs as public goods

#### Communities make more vesicles

If bEVs can carry out different functions depending upon developmental stage, then it stands to reason that vesicles from different species could also carry out different and complementary functions. This concept becomes much more interesting when considering multispecies biofilms. We have already discussed that biofilm bacteria typically produce more OMVs than those in planktonic culture. This has been observed in natural biofilms and in the laboratory (Schooling and Beveridge 2006, Palsdottir et al. 2009, Mozaheb et al. 2022). Our work with PQS-induced OMVs suggests that this phenomenon might arise in multi-species biofilms due to reciprocal stimulation of vesicle biogenesis as a result of signals carried in each species’ respective vesicles. We showed that the *P. aeruginosa* OMV inducing molecule PQS could also stimulate OMV formation and PQS repackaging in a series of other gamma-proteobacterial species (Horspool and Schertzer 2018). The versatility of PQS as a vesicle inducer has also been reported by Tashiro, Ichikawa, Nakajima-Kambe et al. (2010). An intriguing additional finding in our study was that spent supernatant from *E. coli* and *Klebsiella pneumoniae* were also capable of stimulating OMV formation when given to a PQS-null mutant of *P. aeruginosa* (Horspool and Schertzer 2018). Since that time, we have developed systems to harvest and characterize the functions of multispecies OMVs, while also demonstrating that several multispecies pairs hypervesiculate together in biofilms just as well as they do in planktonic culture (unpublished observations). Whether the result of cooperation or competition, these discoveries open the door to the idea that hypervesiculation in the presence of cues from non-self organisms might expand the functionality of community bEVs when bacteria grow in multi-species biofilms.

#### bEVs in the biofilm matrix

**Figure 5:**
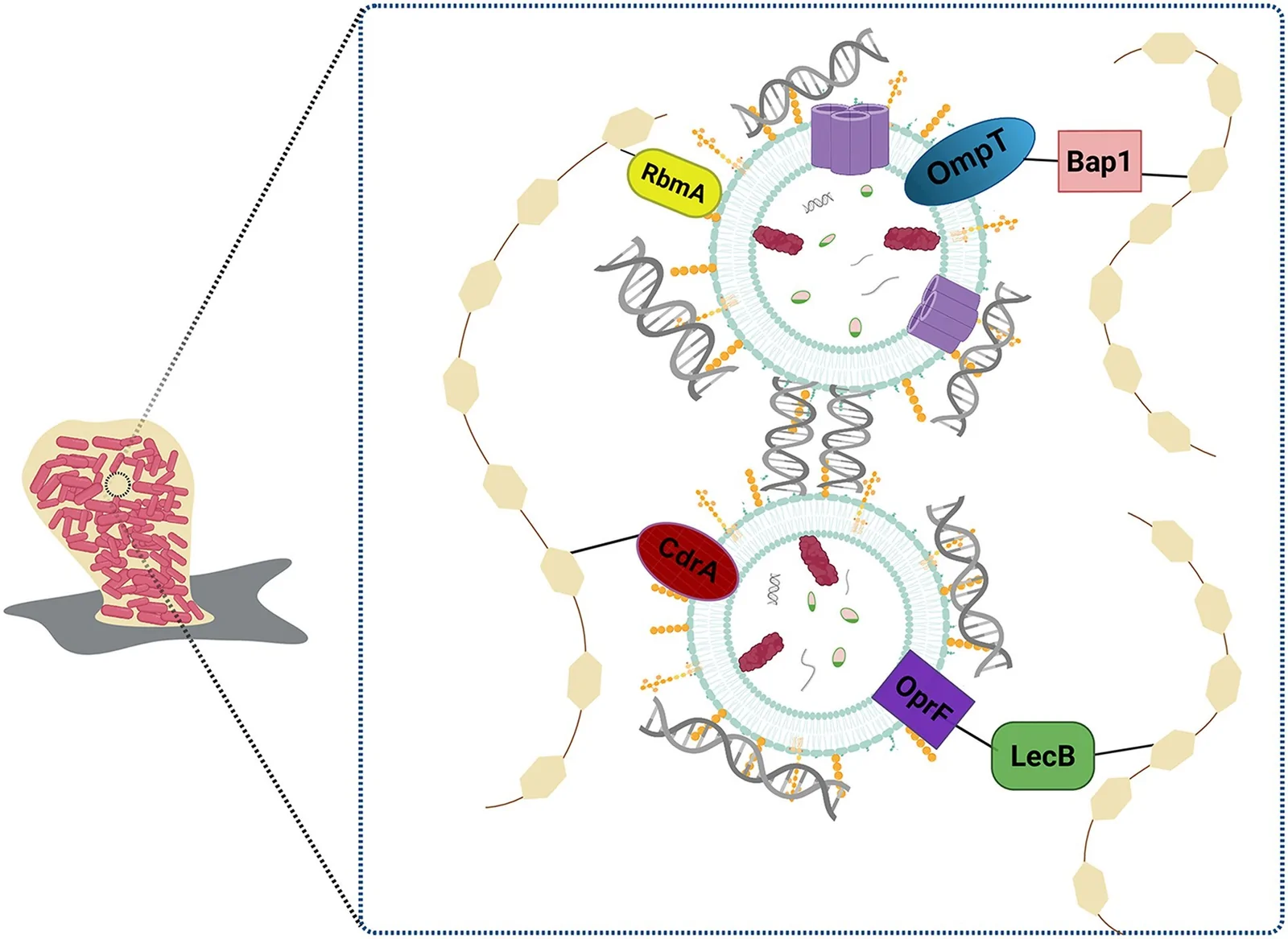
bEVs interact with the biofilm matrix. bEVs make up a significant portion of the biofilm matrix and have been shown to interact with other matrix components. eDNA associates with the surface of bEVs through direct chemical interactions. bEVs interact with polysaccharides through various direct and indirect protein-sugar interactions. The versatility with which bEVs interact with matrix components has led to the suggestion that they perform multiple structural and maintenance functions. Created using biorender.com.

One important biofilm function that has been associated with bEVs is the development and management of the extracellular matrix (Fig. 5). This is a natural connection to make because, even though they are not as well studied as polysaccharides or eDNA, lipids and bEVs make up a substantial portion of the biofilm matrix (Schooling and Beveridge 2006, Palsdottir et al. 2009). Proteomic studies of the matrix have proliferated and they suggest that up to 30% of total matrix protein content might be associated with bEVs (Toyofuku et al. 2012, Couto et al. 2015, Egorova et al. 2022). Because of this, interest has grown to understand how bEVs interact with the components of EPS in the matrix.

The first matrix component that was connected to bEVs was extracellular DNA (Dorward et al. 1989). It was common to test for DNA in early characterizations of purified bEVs (Dorward and Garon 1990, Kadurugamuwa and Beveridge 1995, Kolling and Matthews 1999), and bEV-associated DNA was often found to be bound to the exterior surface of the vesicles (Schooling et al. 2009, Bitto et al. 2017). bEVs may be more involved with biofilm eDNA than simply providing a binding parter, however. Several studies, in both Gram-negative and Gram-positive organisms, have suggested that the process for eDNA release and bEV formation are related, if not the same (Whitchurch et al. 2002, Allesen-Holm et al. 2006, Grande et al. 2015, Toyofuku, Cárcamo-Oyarce et al. 2017, Zhao et al. 2025).

Interactions between bEVs and polysaccharides in the matrix are thought to depend more on specific vesicle-associated proteins. CdrA is an adhesin protein capable of binding both Psl and Pel polysaccharides in the matrix of *P. aeruginosa* biofilms (Reichhardt et al. 2020). Responsive to cyclic-di-GMP control, CdrA can be cleaved from the cell surface by the LapG protease, meaning that CdrA contributes to both cell-associated and cell-free polysaccharide interactions (Rybtke et al. 2015, Reichhardt 2023). It stands to reason that cell-associated CdrA, being integral to the OM through its partner CdrB, would likely be swept up into vesicles as part of the biogenesis mechanism. If that is the case, then CdrA could facilitate direct interaction between OMVs and matrix polysaccharide. Potapova et al. (2024) investigated the ability of *V. cholerae* OMVs to interact with matrix polysaccharides and found that a number of important polysaccharide-binding matrix proteins (Bap1, RbmA, RbmC) associate with OMVs, either directly or indirectly through integral membrane proteins like OmpT. Another example of indirect association involves the Psl-binding lectin LecB of *P. aeruginosa* (Passos da Silva et al. 2019). This lectin can also interact with the major OM protein OprF (Funken et al. 2012). OprF is a major OMV protein in *P. aeruginosa* and therefore provides an avenue for matrix association through LecB (Cassin and Tseng 2019). Our recent finding that OprF exists in a different conformation in OMVs vs. cells (Mathur et al. 2026) suggests that OMVs may bind to the matrix differently than cells do, potentially impacting how vesicles interact with and/or move through the biofilm matrix.

#### bEVs in community metabolism

One area where bEVs have not been well studied in the biofilm context but nevertheless could have a large impact on biofilm ecology is the role of “hunting and gathering” nutrients for embedded biofilm cells. The ability provided by bEVs to leave the cell, process or bind nutrients and return could be a huge advantage. In Bacteroides spp., secreted glycosidases of the Sus family provide the ability to break down complex dietary glycans to provide for bacterial metabolism. Studies using *B. thetaiotaomicron* revealed that acidic glycosidases and SusD-like sugar-binding proteins were associated with OMVs, while the SusC transporter was retained on the cell surface (Elhenawy et al. 2014, Valguarnera et al. 2018). The same group later showed that *B. thetaiotaomicron* can tailor its OMV cargo to secrete specific glycosidases depending upon which glycans are present in the environment (Sartorio et al. 2023). This sets up a system where active OMVs could travel away from cells to liberate and transport sugars back to the producing cell. Similar arrangements exist for peptide and amino acid acquisition. Asaccharolytic *P. gingivalis* must ferment amino acids acquired from the environment for energy. To aid in this, large numbers of gingipain proteases are packaged into OMVs, which then disperse to degrade proteins from the host (Guo et al. 2010, Haurat et al. 2011).

Iron is another critical nutrient that is often limiting in bacterial environments. In addition to freeing up peptides and amino acids, gingipains loaded into *P. gingivalis* OMVs can also degrade hemoglobin, transferrin, and other proteins to liberate both iron and heme, which the bacterium requires (Guo et al. 2010). In contrast, *P. aeruginosa* takes advantage of the iron chelating ability of PQS (Diggle et al. 2007) to incorporate OMVs into its iron scavenging system (Lin et al. 2017). This organism releases the T6SS effector protein TseF to interact with iron-bound PQS on the surface of OMVs. TseF then helps deliver iron-loaded OMVs to interact with siderophore receptor FptA and the OprF porin on the surface of the bacterium. Iron is then released and transported into the cell by an as-yet-unidentified system. In a wild example of metabolic protection, *Shewanella oneidensis* has been reported to shed OMVs as sacrificial membrane to mitigate uraninite surface encrustation of the producing cell while reducing environmental uranium (Shao et al. 2014).

Though these systems have so far been studied only under planktonic conditions, their potential impact on biofilm community metabolism is great, especially in mixed-species biofilms.

#### bEVs in community policing

The final area we will explore is the role bEVs have in controlling the makeup and operation of the biofilm: what gets in, where the population goes, and how information and resources are shared. Palsdottir et al. (2009) beautifully imaged *M. xanthus* biofilms and noted the very non-random distribution of OMVs within them. Notably, the OMVs were concentrated at the periphery, or biofilm-air boundary. This physical location sets the stage for the potential ability of biofilm bEVs to gatekeep what is allowed into the community. In *P. aeruginosa*, Bagge et al. (2004) noted that *ampC* beta-lactamase expression was stimulated at the periphery of biofilms exposed to beta-lactam antibiotics. It is known that β-lactamases are packaged into OMVs in *P. aeruginosa* (Ciofu et al. 2000, Bomberger et al. 2009) and other species (Schaar et al. 2011), and can protect cultures from antibiotic exposure. Thus, the spatially controlled stimulation of protective OMV cargo indirectly supports the idea of OMVs as biofilm gatekeepers. In a related study, Morales-Soto et al. (2018) reported that the edge of *P. aeruginosa* biofilms nearest to antibiotic exposure saw a localized increase in PQS production. Since PQS induces OMV production (Mashburn and Whiteley 2005, Schertzer and Whiteley 2012) and the increase of one correlates with the increase of the other in biofilms (Cooke et al. 2019, 2020), this provides more indirect evidence of OMV gatekeeping. Following along with the theme, *P. aeruginosa* biofilms increased PQS, and likely OMV, production only in regions coming into close proximity to other bacteria (Cao et al. 2020). A separate study also showed that phage-infected *P. aeruginosa* biofilms overproduced PQS, and likely OMVs, which then repelled phage and incoming uninfected *P. aeruginosa* swarms (Bru et al. 2019).

In mobile biofilm communities like those of *M. xanthus*, it appears OMVs play an important role in directing the traffic. The Stigmergic movement of *M. xanthus* swarms is facilitated by trail-following behavior where leading cells deposit slime that makes it easier for subsequent cells to follow in the same path. Ducret et al. (2013) used single-cell imaging techniques to characterize this movement and discovered that cells shed significant membrane in the form of OMVs and membrane tubes into the slime trail. It was speculated that following cells could “pick up” the OMVs in the trail by membrane fusion to recycle OM and receive signals from the preceding cells. Gloag et al. (2016) conducted a similar study and were able to image >100 nm sized OMVs in the slime trail by super resolution microscopy. They further speculated that the deposited membrane material could “lubricate” cellular movement along the trail.

Finally, OMVs also play a key role in facilitating important exchanges between biofilm cells. Horizontal gene transfer has been a well-accepted function of bEVs in planktonic settings for some time (see “communication” above). Johnston et al. (2023) recently demonstrated that biofilm OMVs could also transfer antibiotic resistance plasmids in *P. aeruginosa*. The authors noted that OMVs purified from biofilms harbored more DNA than planktonic OMVs and were more effective at DNA exchange than either planktonic OMVs or naked plasmid. In addition to sharing DNA, bacteria in a community can swap membrane in order to modify their cell surfaces. Zingl et al. (2020) used hypervesiculating VacJ/Yrb mutant *V. cholerae* strains to investigate the effect that increased surface exchange would have on bacterial adaptation. They found that increased surface exchange facilitated by the hypervesiculating phenotype allowed strains to quickly accumulate glycine-modified LPS and deplete the OM porin OmpT. These adaptations conferred resistance to antimicrobial peptides and bile, respectively. Thus, OMV-mediated surface exchange resulted in changes that would allow increased intestinal colonization fitness. In the Gram-positive organism *Bacillus subtilis*, Tzipilevich et al. (2017) described a similar phenomenon, where phage resistant cells exposed to bEVs from a phage sensitive strain would acquire temporary phage sensitivity due to transient display of the phage receptor as a result of surface exchange from bEV fusion. This is in contrast to the work of Augustyniak et al. (2022) described above, who were unable to detect transfer of phage receptors by bEVs.

## Concluding remarks

Across all domains of life, it has become clear that extracellular vesicular trafficking plays an important role in intercellular interactions. The focus of this review has been on bEVs, their biogenesis, and their function—with particular emphasis on how they shape bacterial communities. Although a relatively new field, much has been learned from planktonic studies about vesicle formation and function. Nevertheless, a universal consensus model describing how bEVs are produced remains elusive. Important differences in the physiology, environment, and behavior of bacterial species appear to influence how they generate vesicles. If we consider that bacterial EV production may showcase early evolutionary attempts to solve the vesicle secretion problem using simple systems that must operate, at least for Gram-negative organisms, in a cellular compartment lacking easy access to reducing potential or ATP, we may have to entertain the possibility that there will be no universally conserved mechanism. Instead, each species may have had to develop overlapping systems that take advantage of broad principles like stress response, Bilayer-Couple style membrane bending or envelope component recycling. Because of this, it may be most informative to categorize biogenesis mechanisms into general classes, as we have tried to do here, and observe how those principles inform vesicle production throughout the tree of life. An exciting open question is whether bacterial vesicle biogenesis systems directly gave rise to eukaryotic systems, and how such an evolution may have taken place.

The bulk of studies on bEVs have been performed using Gram-negative organisms grown planktonically. This has generated an invaluable knowledge base, but the field is poised to explore new frontiers. First, support is growing for the idea that Gram-positive bEVs are more than just cellular debris, and that these organisms make use of vesicle secretion for purposes similar to Gram-negative organisms. This shift in thinking will require a re-evaluation and expansion of how we conceptualize vesicle formation, and this may feedback into the study of cell lysis mechanisms in Gram-negative bacteria. A bigger leap is poised to take place as we transition from single-species planktonic studies to observing and testing vesicle secretion in multi-species and biofilm communities. Similar to how intracellular and extracellular vesicles make such major contributions to the biology of multicellular eukaryotic organisms, we will also likely see the greatest impacts of bEVs on diverse, spatially organized bacterial communities. The potential for bEVs to impact community organization and management through the glimpses we’ve already had into biofilm communication, matrix remodeling, horizontal gene transfer, surface sharing, and community defense is truly exciting. New challenges remain, however. There are seeming contradictions encountered when trying to determine if bEVs promote or hinder biofilm growth. Biofilm bEV proteomics studies are also likely to conflict or cause confusion when results are compared between studies. New discoveries regarding the dynamic nature of bEV number, content, and function depending upon time of production and geography within a biofilm help to explain this. Going forward, researchers will have to take care to understand the context from which bEVs were harvested when analyzing their functions and effects.

Despite new challenges and ever more complex systems, the future of the bEV field continues to look bright. A thorough understanding of vesicle formation and function in bacterial communities will be critical not only to our understanding of bacterial physiology and ecology, but also to the exploitation of bEVs in downstream applications such as vaccine development or targeted antimicrobial drug delivery.

## Data Availability

This review article discusses the work of many authors in the topic area. We did not present new data here. Please consult the citations within the text to find source data relating to the topics discussed.
